# Effects of two different strategies of fluid administration on inflammatory mediators, plasma electrolytes and acid/base disorders in patients undergoing major abdominal surgery: a randomized double blind study

**DOI:** 10.1186/1476-9255-10-29

**Published:** 2013-09-24

**Authors:** Carlo Alberto Volta, Alessandro Trentini, Lucia Farabegoli, Maria Cristina Manfrinato, Valentina Alvisi, Franco Dallocchio, Elisabetta Marangoni, Raffaele Alvisi, Tiziana Bellini

**Affiliations:** 1 Department of Surgical, Anaesthesiological and Radiological Science, Section of Anaesthesia and Intensive Care, S. Anna Hospital, University of Ferrara, Via Aldo Moro n. 8-44124 Cona, Ferrara, Italy; 2 Department of Biochemistry and Molecular Biology, Section of Biochemistry and Clinical Biochemistry, University of Ferrara, via Luigi Borsari 46, Ferrara 44121, Italy

**Keywords:** Balanced solutions, Anti-inflammatory mechanisms, Plasmatic electrolytes, pH, Kidney function

## Abstract

**Background:**

Administration of normal saline might increase circulating levels of pro-inflammatory cytokines and may cause variation of plasmatic electrolytic and hyperchloremic acidosis, which in turn can impair renal function. Hence the use of balanced solutions could influence the inflammatory cascade triggered by the surgical procedures, the plasmatic electrolyte concentration, the acid–base equilibrium, and the renal function.

**Methods:**

This is a double blind randomized trial. Forty patients undergoing major abdominal surgery (bowel cancer) were allocated in two groups, the balanced solution (BS) group in which the fluids administered were balanced solutions (colloids and crystalloids); and the unbalanced solution (UBS) group in which the fluids administered were unbalanced solutions (colloids and crystalloids). Measurements were performed after anaesthesia induction (T0), at the end of surgery (T1), within 2 h after surgery (T2) and 24 h after the beginning of surgery (T3). The following data were collected: 1) active matrix metalloproteinase 9 (MMP-9) and its tissue inhibitor (TIMP-1), IL-6, IL-8, IL-10; 2) blood gases variables; 3) electrolytes, albumin, total serum protein and the strong ion difference; 4) neutrophil gelatinase-associated lipocalin (NGAL) from urinary sample.

**Results:**

The BS group exhibited higher circulating level of IL-10 and TIMP-1 and lower level of active MMP-9. The UBS group experienced hypercloremia, hypocalcemia, hypomagnesemia, worse acid–base equilibrium and higher level of NGAL.

**Conclusions:**

The use of balanced solutions was responsible of less alteration of plasmatic electrolytes, acid–base equilibrium, kidney function and it might be associated with an early anti-inflammatory mechanisms triggering.

**Trial registration:**

ClinicalTrials.gov (Ref: NCT01320891).

## Background

Fluid therapy plays a fundamental role in the management of patients undergoing major surgery. Hypovolemia can cause organ dysfunction unless an adequate fluid resuscitation volume is provided. However, it is not only a question of the quantity of fluids administered, quality must also play an equally fundamental role. Administration of 0.9% saline can be harmful because it can cause hyperosmolar states [1] and hyperchloremic acidosis [2-5]. Nevertheless, little is known about the possible variation of plasmatic levels of other electrolytes than Na^+^ and Cl^-^, induced by normal saline administration. This because both acidosis and the dilution of plasma due to a administration of solutions free of Ca^2+^ and Mg^2+^ might influence their plasmatic concentration. Furthermore, administration of normal saline decreased glomerular filtration rate and urinary output [4,6]. Of note, normal saline administration can lead to oedema in the gastrointestinal tract, an increase in abdominal pressure, and reduced renal perfusion [7,8]. Finally, when compared with balanced solutions, administration of normal saline increases the circulating levels of pro-inflammatory cytokines in septic shock [9]. Hence it can hypothesised that the use of balanced solutions can influence the inflammatory cascade triggered by the surgical procedures. This should be particularly relevant during major intestinal surgery since such surgery is associated with more inflammatory response than non-intestinal minor surgery [10]. The pro-inflammatory cytokines trigger the release of various substances. A significant role is played here by matrix metalloproteinase 9 (MMP-9), since its over-production is associated with anastomotic leakage after bowel surgery [11]. Hence the aim of our study was to verify if the use of balanced solutions can influence 1) the inflammatory cascade and specifically the activity of MMP-9 and its natural inhibitor (TIMP-1); 2) the electrolytes plasmatic levels, acid –base equilibrium and renal function.

## Methods

The study was approved by the ethics committee of the S. Anna University Hospital, Ferrara, Italy (Ref: 28/1/10) and registered with ClinicalTrials.gov (Ref: NCT01320891). Informed consent was obtained from all subject prior to the randomization.

### Participants

This was a double blind randomized trial. Eligible patients were those aged 18 years or older, undergoing abdominal surgery for bowel cancer. Exclusion criteria were: 1) emergency surgery for bowel punch or intestinal occlusion; 2) cardiac failure (New York Heart Association class III or IV); 3) kidney dysfunction (serum creatinine > 200 μmol/L); 4) preoperative anemia (hemoglobin < 10 g/dl); 5) therapy with corticosteroids or nonsteroidal anti-inflammatory substances; 6) allergy to hydroxyethyl starches and 7) patient rejection of participation in the study.

Patients were randomly allocated in a 1:1 ratio in two groups, the balanced solution (BS) group in which the fluids administered were always balanced solutions, both as colloids and crystalloids; and the unbalanced solution (UBS) group in which the fluids administered were always unbalanced solutions, both as colloids and crystalloids. The randomization list was created by using a computer based block randomization by a statistician prior to the initiation of the study. Patients fulfilling the inclusion criteria received an increasing sequential number in accordance with the order of their randomization and inclusion in the study. The allocation concealment was ensured by pharmacy-controlled randomization: on the basis of the randomization list, the pharmacies distributed patient-specific colloid and crystalloid bottle that were identical in size, weight and appearance. Patients allocated to BS group were treated with colloid bottles containing 6% HES 130/0.42 in plasma adapted Ringer’s acetate/malate solution (Tetraspan®; B.Braun Melsungen, Germany) and crystalloid bottles containing plasma adapted Ringer’s acetate/malate solution (Sterofundin® ISO; B.Braun, Germany). Patients allocated in UBS group received colloid bottles containing 6% HES 130/0.42 diluted in unbalanced normal saline solution (Amidolite®; B.Braun, Germany) and crystalloid bottles containing normal saline solution (NaCl 0.9%, Fresenius Kabi, Germany). All study investigators, staff members and patients were blinded to the study medication.

Volume administration was started after induction of anaesthesia and continued until 8 a.m. of the first postoperative day. A ratio of 3:1 between crystalloids and colloids was used in both groups. As part of our routine management of fluid administration, the quantity was based on maintaining a mean arterial pressure (MAP) of at least 60 mmHg and a central venous pressure (CVP) of about 10–11 mmHg during surgery.

Till 24 h after the beginning of surgery, fluid administration continued using 1500–2000 ml of respectively unbalanced solution in the UBS group and balanced solution in the BS group. During the entire study period, packed erythrocytes were given when hemoglobin was less than 7 g/dl in patients with normal cardiac function or less than 9 g/dl in patients with ischemic heart disease.

Induction of anaesthesia was performed with propofol (2 mg/kg), fentanyl (3 μg/kg) and vecuronium (0.1 mg/kg) for neuromuscular blockade. Anaesthesia was maintained with fentanyl (2–3 μg/kg/h), sevoflurane (at least 1 MAC and vecuronium, titrated according to the patients’ needs; or with a continuous infusion of propofol, remifentanyl, vecuronium or cisatracurium at weight related doses. Nonsteroidal anti-inflammatory drugs were not administered throughout the investigation period. A warming cover blanket system and fluid warmers were used to avoid hypothermia during surgery.

### Measurements

Perioperative monitoring included measurement of the electrocardiogram, arterial blood pressure, central venous pressure, oxygen saturation and end tidal CO_2_.

Measurements of clinical variables were performed immediately after anaesthesia induction and before volume administration (T0), at the end of surgery (T1), within 2 h after surgery (T2) and 24 h after the beginning of surgery (T3). The following data were collected: 1) blood gases variables, including lactate concentrations from arterial and venous blood samples; 2) electrolytes, albumin and total serum protein from venous blood sample; the strong ion difference (SID) was determined as previously described [12]; 3) neutrophil gelatinase-associated lipocalin (NGAL) from urinary sample; 4) MMP-9 total and active, TIMP-1, IL-6, IL-8, IL-10 from venous blood samples; the MMP-9/TIMP-1 ratio was calculated as an index of equilibrium between the action of MMP-9 and its inhibition.

### IL-6, IL-8 and IL-10 detection assay

IL-6, IL-8 and IL-10 levels were simultaneously measured in sera of patients, diluted 1:2 with dilution buffer, by a multiplex sandwich enzyme-linked immunosorbent assay (ELISA) system based on chemiluminescence detection (Aushon SearchLight chemiluminescent array kits; Tema Ricerca, Italy) according to the manufacturer’s recommendations. The interleukin levels are reported as pg/ml. The detection limits were 0.19 pg/ml (IL-6), 0.39 pg/ml (IL-8) and 0.39 pg/ml (IL-10).

### MMP-9 and TIMP-1 detection assay

Peripheral blood samples were collected in anticoagulant-free test tubes and kept in ice until centrifugation at 3,000 rpm for 10 min. Serum samples were stored in aliquots at −80°C until assay. Serum concentrations of active and total (active + inactive) MMP-9 were measured by using a commercially available activity assay system kit (GE Healthcare, RPN2634), as previously described in details [13]. Serum levels of TIMP-1 were measured by using a commercially available “sandwich” enzyme-linked immunosorbent assay kit (GE Healthcare, RPN2611) as previously described in details [13].

### NGAL detection assay

The urinary NGAL was determined by using the ArchitectR analyzer (Abbott Diagnostics, Illinois, USA).

### Statistical analysis

To detect a difference in MMP-9/TIMP-1 ratio of 0.15 with an SD of 0.14, with a type I error of 0.05 and a power of 0.80, 30 patients should have had to be recruited, 15 in each group. Considering a dropout rate of 20% and in view of the limited comparability of the study condition, the sample size calculation resulted in *n* = 2 × 20 patients. The approximate degree of normal distribution was calculated for each parameter by the Kolmogorov-Smirnov test. Comparisons for continuous variables within and between groups were performed with the Friedman repeated-measures analysis of variance on ranks. To isolate divergent variables, multiple comparison procedures were used (Dunnett’s Method). A P value of less than 0.05 was accepted as statistically significant.

## Results

Patients did not differ with regard to demographic and biometric data, duration of surgery and quantity of fluids administered (Table 1).

**Table 1:** Clinical characteristics and data from peri-operative period

	**UBS**	**BS**
	**(n = 20)**	**(n = 20)**
Age (years)	69 ± 15	68 ± 10
Men/Women	8/14	11/9
Weight (Kg)	71 ± 9	76 ± 10
Height (cm)	166 ± 7	169 ± 7
BMI (Kg/m^2^)	26 ± 3	26 ± 4
ASA Physical Status (I/II/III)	2/10/10	1/11/8
Duration of surgery (min)	245 ± 52	268 ± 80
Infused cristalloid solution (ml)	2920 ± 651	3325 ± 763
Infused colloidal solution (ml)	857 ± 226	883 ± 285

UBS = Un-Balanced Solution group; BS = Balanced Solution group.

ASA = American Society of anesthesiologists classification.

Results are expressed as mean ± SD.

No differences in terms of hemodynamic data were detected among the two groups (Table 2). Six patients in UBS group and two patients in BS group received one or two units of packed erythrocytes.

**Table 2:** Peri-operative clinical parameters

		**HR (min**^**-1**^**)**	**MAP (mmHg)**	**CVP (mmHg)**	**Diuresis (ml)**	**Na**^**+ **^**(mmol/L)**	**K**^**+ **^**(mmol/L)**	**P**^**-**^** (mmol/L)**	**Alb**^**-**^** (mmol/L)**	**PaCO**_**2 **_**(mmHg)**
UBS	T0	68 ± 9	75 ± 13	14 ± 3	71 ± 79	142 ± 2,1	4,8 ± 1,3	5,6 ± 1,1	0,84 ± 0,1	38,2 ± 4,4
	T1	70 ± 10	79 ±10	15 ± 3	586 ± 366	143 ± 2,8	4,6 ± 1,2	5,5 ± 1,2	0,63 ± 0,1^§^	40,7 ± 3,9^§^
	T2	78 ± 13	94 ± 10	13 ± 4	841 ± 407	143 ± 2,0	4,6 ± 1,3	5,7 ± 1,0	0,65 ± 0,1^§^	44,2 ± 5,8^§^
	T3	82 ± 15	88 ± 7	9 ± 4	2230 ± 878	143 ± 3,7	4,5 ± 1,14	5,0 ± 1,2	0,71 ± 0,1^§^	38,5 ± 4,5
BS	T0	62 ± 12	87 ± 17*	14 ± 4	146 ± 181	142 ± 2,5	5.0 ± 0,7	5,5 ± 0,9	0,77 ± 0,4	37,6 ± 3,4
	T1	65 ± 10	78 ± 9	15 ± 5	690 ± 434	142 ± 1,8	4,8 ± 0,6	6,7 ± 1,8*^§^	0,68 ± 0,3	41,4 ± 3,8^§^
	T2	73 ± 17	92 ± 13	13 ± 5	1005 ± 678	142 ± 1,8	4,9 ± 0,6	7,1 ± 1,7*^§^	0,57 ± 0,3	43,5 ± 5^§^
	T3	83 ± 10	91 ± 14	8 ± 5	2533 ± 1076	141 ± 3,1	4,1 ± 0,6^§^	5,5 ± 1,7	0,62 ± 0,4	41,1 ± 4,8^§^

UBS = Un-Balanced Solution group; BS = Balanced Solution group; HR = Heart rate; MAP = mean arterial pressure; CVP = central venous pressure; NGAL = neutrophil gelatinase-associated lipocalin.

Results are expressed as mean ± SD.

* *P* < 0.05 comparison made between the two groups.

§ *P* < 0.05 comparison made within each group having T0 as a control value.

### Inflammatory response

**Figure 1:**
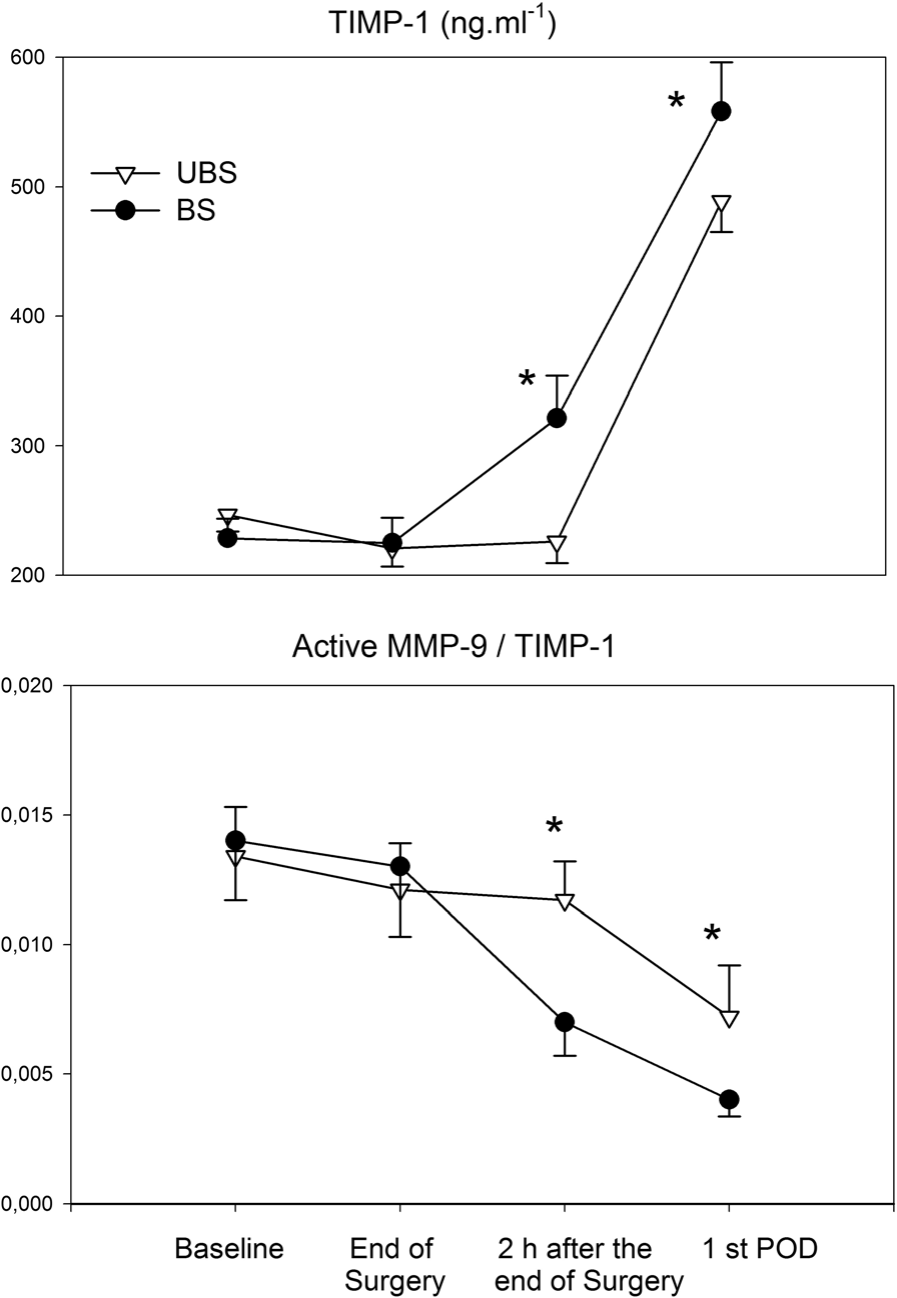
**Changes of tissue specific inhibitor of metalloproteinases (TIMP-1) and the ratio between matrix metalloproteinase – 9 (MMP-9) and TIMP-1.** POD: postoperative day. Data are presented as mean ± SD. * p < 0.01.

IL-6 and IL-8 increased similarly in both groups (Table 3), whilst IL-10 was higher in the BS group (Table 3). The ratio between active MMP-9 and TIMP-1 (MMP-9/TIMP-1) was statistically lower in the BS group (Figure 1). This was due to a lower plasmatic level of active MMP-9 and a higher plasmatic concentration of TIMP-1 in the BS group (Figure 1).

**Table 3:** Plasmatic inflammatory mediators

		IL – 6 (pg*ml^**-1**^**)**	IL – 8 (pg*ml^**-1**^**)**	IL- 10 (pg*ml^**-1**^**)**
**UBS**	**T0**	5.6 [3.2 - 13.3]	2.9 [1.3 – 4.8]	1.3 [0.9 – 2.6]
	**T1**	146 [83–243]	14.5 [5.3 - 18]	16.8 [9.1 – 31.1]
	**T2**	391.1 [196–631]	21.3 [8.5 – 31.7]	24.9 [13.5 – 56.1]
	**T3**	131.9 [106–179]	11.2 [4.4 – 14.9]	6.8 [4.5 – 14.1]
**BS**	**T0**	4.9 [2.8 - 7.4]	2.5 [1.6 – 3.9]	1.2 [1.0 – 1.8]
	**T1**	208.8 [59–391]	12.3 [4.5 – 21.8]	30.6* [15.2 – 49.3]
	**T2**	430.6 [149–539]	28.2 [12.6 – 34.1]	41.1* [19.3 – 63.8]
	**T3**	138 [98–178]	12.6 [8.9 – 17.6]	14.2* [7.0 – 18.2]

UBS = Un-Balanced Solution group; BS = Balanced Solution group.

Results are expressed as median and 25th -75th percentile for IL-6 and IL-8; as mean ± SD for active MMP-9. * *P* < 0.01.

### Electrolytes and acid–base disorders

**Figure 2:**
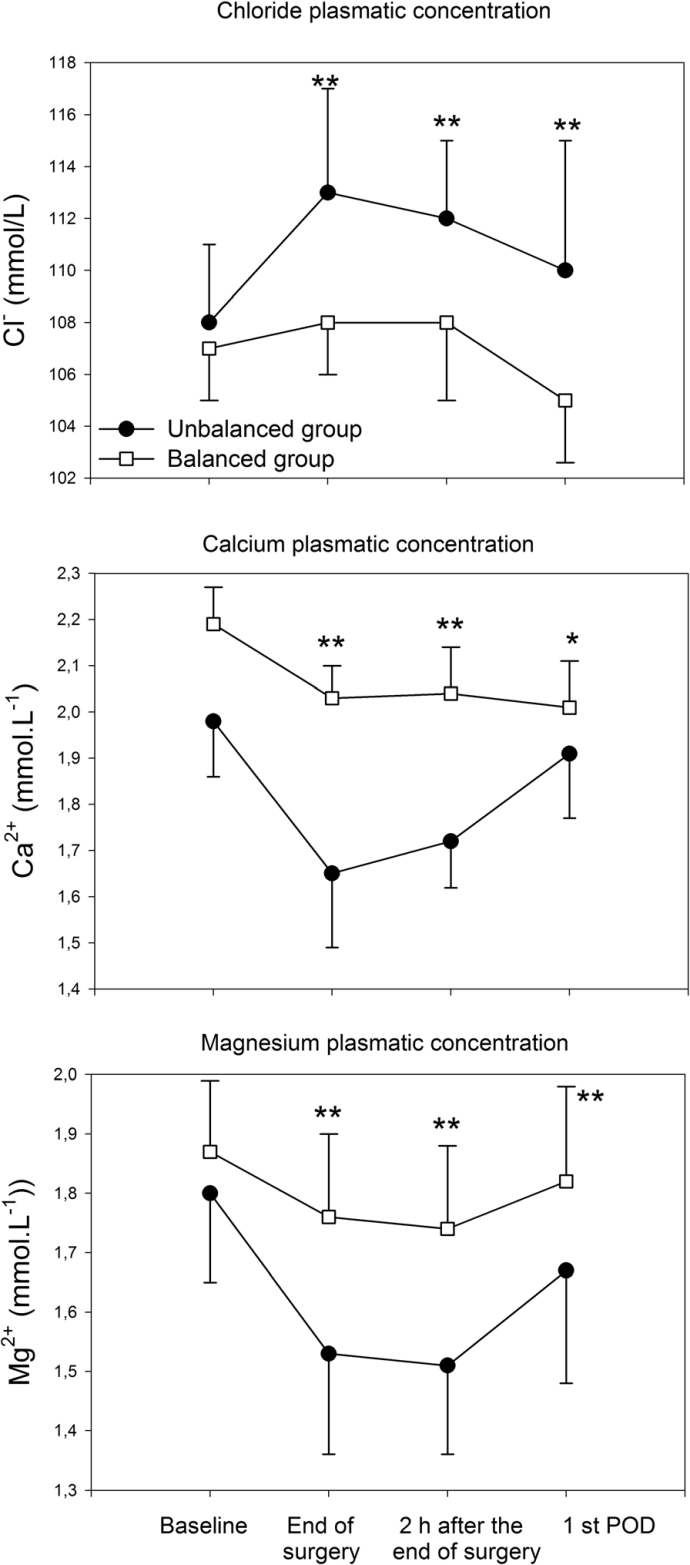
**Changes of Chloride, Calcium and Magnesium plasma levels.** Note that 38% of the patients of the UBS group received Calcium after surgery. POD: postoperative day. Data are presented as mean ± SD. * p <0.05 and ** p<0.01.

**Figure 3:**
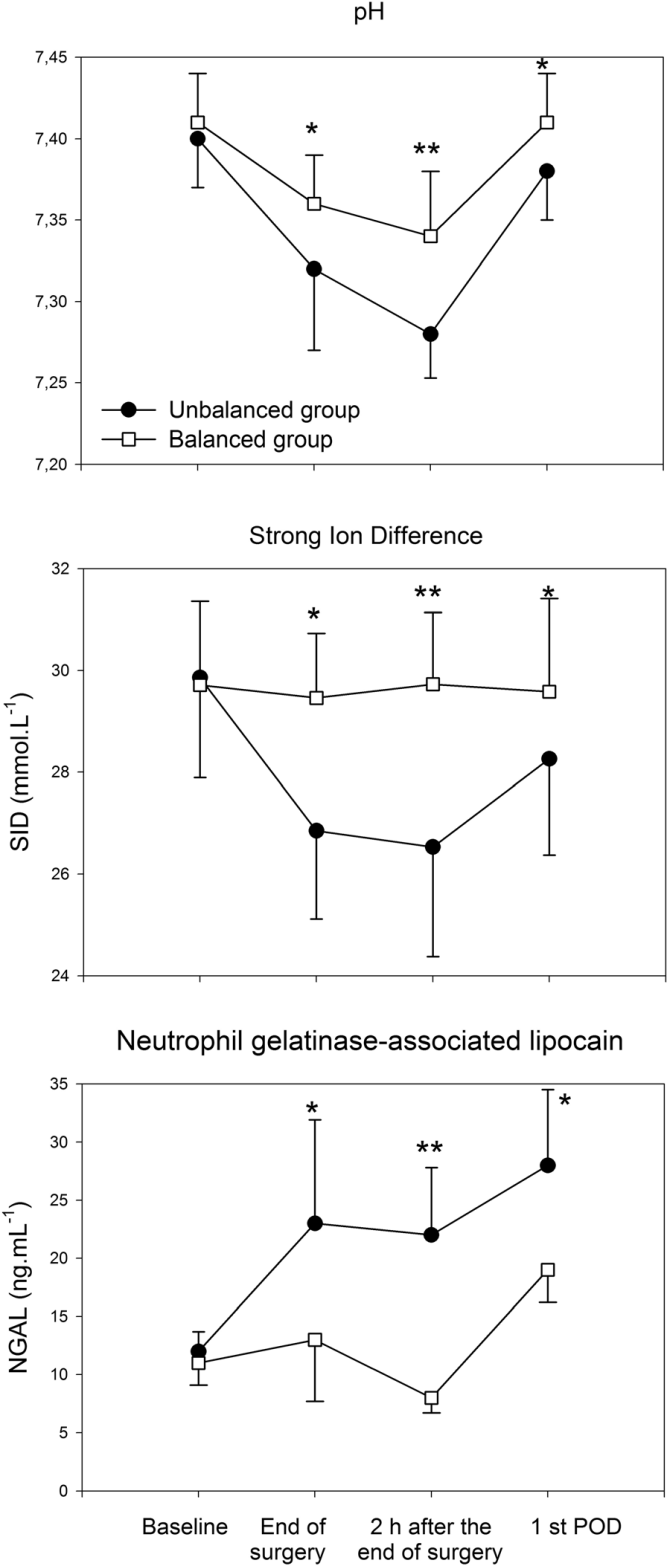
**Changes of pH (panel A), Strong Ion Difference (panel B) and neutrophil gelatinase-associated lipocalin (panel C).** POD: postoperative day. Data are presented as mean ± SD for pH and SID; as mean ± SE for NGAL. * p <0.05 and ** p<0.01.

The main changes in electrolyte concentrations are presented in Table 2 and Figure 2. The two groups differed in terms of Cl^-^, Ca^2+^, Mg^2+^ and Phosphate. More specifically, the UBS group experienced higher plasmatic concentration of Cl^-^ and lower concentrations of Ca^2+^ and Mg^2+^ and P^-^ (Table 2 and Figure 3). Of note, 38% of the patients of the UBS group received Calcium after surgery because of its low plasmatic level, thus influencing the value of Ca^2+^ of the UBS at the first postoperative day (Figure 2). Furthermore the UBS group exhibited lower values of pH (Figure 3) in presence of similar values of PaCO_2_ (Table 2). HCO_3_ ^-^ and BE valves were significantly lower in the UBS group (data not shown). Similarly the values of SID were statistically higher in BS group (Figure 3).

### Renal function

Whilst the urine output was similar among the two groups throughout the study (Table 2), the NGAL urinary concentration in the BS group were statistically lower compared to the value found in UBS group (Figure 3).

## Discussion

The main findings of our study are that a fluid strategy based on administration of balanced solutions both as crystalloids and colloids is associated with: 1) increased production of anti-inflammatory cytokines and less expression of active MMP-9; 2) less variation of plasmatic electrolytes and pH; 3) less increase of NGAL.

### Inflammatory response

Surgical procedures are associated with a great and sharp increase of pro-inflammatory cytokines [14]. Kellum et al. [9]. in a septic animal study showed that the use of a balanced fluid replacement strategy was associated with less expression of pro-inflammatory cytokines; in contrast, hyperchloremic acidosis worsened hemodynamic variables and increased circulating inflammatory molecules in an animal model of septic shock [15]. We cannot confirm this result in patients undergoing major abdominal surgery since there was no statistical difference between the two groups in terms of circulating pro-inflammatory cytokines concentration, such as IL-6 and IL-8 (Table 3). However, the anti-inflammatory cytokine (IL-10) was statistically higher in the BS group (Table 3), underlying that the use of balanced solutions could be associated with faster recovery of the disequilibrium between pro and contra- inflammatory cytokines balance.

In this connection, it should be pointed out that the over-expression of pro-inflammatory cytokines is associated with an increased plasmatic level of MMP-9, the role of which has been previously investigated. During an acute inflammatory response, excessive production of MMP-9 is linked to tissue damage and degenerative inflammatory disorders [16-19]. Major abdominal surgery is responsible for an over-expression of MMP-9 as part of the inflammatory process triggered by the surgical procedure [13]. Stumpf et al [11]. demonstrated a significantly greater expression of MMPs in mucosal and submucosal layers of patients with anastomotic leakage after large bowel surgery compared with patients with uncomplicated anastomotic healing. Hence procedures decreasing circulating levels of active MMP-9 could play a fundamental role in the perioperative management of patients undergoing major abdominal surgery. We have previously demonstrated that administration of HES 130/0.4 in patients undergoing major abdominal surgery was able to decrease the plasmatic concentration of MMP-9 with no significant effect on its natural inhibitor, i.e. TIMP-1. The net result was the reduction of the activity of MMP-9 [13]. The same result was obtained in the present study by using balanced solutions. Indeed, the ratio between the active MMP-9 and TIMP-1 was more favourable in the BS group (Figure 1). This result was not only due to a decreased plasmatic concentration of active MMP-9, but also to a higher concentration of TIMP-1 in the BS group. It is tempting to hypothesize that, as for IL-10, the contra inflammatory mechanisms seems to start earlier in patients treated with balanced solutions.

### Electrolytes and acid –base equilibrium

A strategy of fluid replacement based on normal saline both in terms of crystalloids and colloids was associated with a lower pH and a lower concentration of bicarbonate and BE (data not shown). The strong ion difference followed these variations being statistically lower in the UBS group (Figure 3). Indeed normal saline contains no bicarbonate precursor, such as lactate, malate or bicarbonate. Hence normal saline administration should dilute the bicarbonate concentration of the extracellular space. Based on the Stewart’s approach, the decrease of the strong ion difference is mainly the result of the plasmatic increase of chloride (hyperchloremic acidosis), as it was the case in the present study (Figure 3). Furthermore the use of balanced solutions was associated with more physiological values of plasmatic electrolytes (Table 2 and Figure 2). Although this was expected for chloride, we were surprised by the low plasmatic level of both calcium and magnesium in the UBS group. Calcium is relevant for excitation-contraction coupling (cardiac arrhythmias), ciliary movements, neurotransmitters release, enzyme secretion, hormonal secretion and coagulation. The latter should be considered carefully in the clinical setting because the clotting activity is influenced mostly by temperature, pH and calcium concentration. This could be relevant in bleeding patients, such as those with major trauma [20]. A recent paper [21] underlines that hypocalcaemia has an inverse linear concentration-dependent relationship with mortality. In this connection, it should be noted that calcium was administered intravenously by the treating surgeon only in the UBS group because of its low plasmatic concentration.

Calcium and magnesium are closely related to each other and clinical manifestation of Mg^2+^ deficit includes tetany, seizures, arrhythmias, neuromuscolar irritability, hypocalcemia and hypokalemia. Magnesium has anti-nociceptive effects since it reduces the need for intraoperative anaesthetics and relaxant drugs and reduces the amount of morphine for the treatment of postoperative pain. Furthermore magnesium modulates cellular events involved in inflammation. Experimental magnesium deficiency in the rat induces a clinical inflammatory syndrome characterized by leukocyte and macrophage activation, release of inflammatory cytokines and acute phase proteins, excessive production of free radicals [22,23].

The mechanisms by which a not balanced strategy of fluid replacement therapy is associated with hypomagnesemia can be related to the absence of Mg^2+^ and by acidosis itself, which promotes intracellular shift of Mg^2+^.

### Renal function

Although diluted in balanced and unbalanced solutions, both groups of patients received the same type and amount of HES 130/0.42. The influence of HES on kidney function remains controversial. Histological studies have demonstrated reversible swelling of tubular cells of the kidneys (‘osmotic nephrosis like-lesions’) [24]. The importance of this finding remains unclear as other substances also may induce this effect. When assessing kidney function with regard to different volume replacement strategies, the composition of the solvent has to be taken into consideration as well. In denervated kidneys, it has been shown that hyperchloremia produced a progressive renal vasoconstriction and fall in GFR that is independent of the renal nerves [25]. In humans, plasma renin activity was suppressed by sodium chloride but not by sodium bicarbonate infusion [26]. As serum Creatinine is influenced by several factors (e.g., muscle mass and age), we measured urinary concentrations of kidney-specific proteins such as NGAL to assess the influence of our volume replacement strategies on tubular integrity [27]. NGAL is upregulated by ischemia in several segments of the nephron, predominantly in proximal tubules and it is suggested to be an early marker of acute renal injury [27,28]. Dent et al [29]. found the 2-hour postoperative NGAL level a reliable predictor of duration of AKI and length of hospital stay while the 12-hour NGAL level was a predictor of mortality. Similarly, Bennett et al [30]. found the 2-hour NGAL a reliable predictor of severity and duration of AKI, length of hospital stay, renal replacement therapy requirement and mortality. We found a lower increase of NGAL in the balanced group (Figure 3) and hence it could be hypothesized that balanced solutions could be protective for renal function. Moreover, using a cut-off value of 100 μg/ml for the 2-hour urinary NGAL concentration, the area under the curve (AUC) was 0.95, sensitivity 82% and specificity 90% for prediction of AKI [30]; since only two patients of the UBS group had urinary NGAL values higher than 100, we can hypothesized that the perioperative use of HES130/0.42 diluted in balanced solution could be considered safe for the renal function in patients with normal preoperative renal function.

## Conclusion

Our study could suggest that the total balanced approach to fluid therapy might be associated with an early anti-inflammatory mechanisms triggering, testified not only by an early increase of IL-10, but also by a more favourable MMP-9/TIMP-1ratio in the BS group. However, given the small number of patients enrolled, further studies are required to clarify the extent of the anti-inflammatory mechanisms and its clinical role. Finally, the group treated with balanced solutions experienced less alteration of plasmatic electrolytes, acid–base equilibrium, and kidney function.

## Abbreviations

MMP-9: Matrix metalloproteinase 9; TIMP-1: Tissue inhibitor of matrix metalloproteinase-1; UBS: Unbalanced solutions; BS: Balanced solutions; HES 130/0.42: hydroxyethyl starches 130/0.42; MAP: Mean arterial pressure; CVP: Central venous pressure; SID: Strong ion difference; NGAL: Neutrophil gelatinase-associated lipocalin; IL-6: Interleukin-6; IL-8: Interleukin-8; IL-10: Interleukin-10.

## Competing interests

B.Braun Italy supported this study by providing the fluids used and by covering all the expense for the dosage of IL-6, IL-8, IL-10, MMP-9 and TIMP-1.

## Authors’ contributions

CAV conceived and designed the study, analyzed part of the data and drafted the manuscript. AT carried out part of the immunoassays and helped to design the study. LF coordinated the study, enrolled and monitored the patients, and analyzed the data. MCM carried out part of the immunoassays. VA helped to study patients, to acquired data, and to draft the manuscript. FD participated in the design and coordination of the study (Biochemical part). EM helped to study patients and performed the statistical analysis. RA participated in the design of the study and helped to draft the manuscript. TB participated in the coordination of the study and helped to draft the manuscript. All authors read and approve the final manuscript.
